# Clinical and genetic spectrum of sarcoglycanopathies in a large cohort of Chinese patients

**DOI:** 10.1186/s13023-019-1021-9

**Published:** 2019-02-14

**Authors:** Zhiying Xie, Yue Hou, Meng Yu, Yilin Liu, Yanbin Fan, Wei Zhang, Zhaoxia Wang, Hui Xiong, Yun Yuan

**Affiliations:** 1 Department of Neurology, Peking University First Hospital, 8 Xishiku St, Xicheng District, Beijing, 100034 China; 2 Department of Pediatrics, Peking University First Hospital, Xishiku St, Xicheng District, Beijing, 100034 China

**Keywords:** Sarcoglycanopathies, Phenotype, Genotype, Sarcoglycan expression

## Abstract

**Background:**

Sarcoglycanopathies comprise four subtypes of autosomal recessive limb-girdle muscular dystrophy (LGMD2C, LGMD2D, LGMD2E, and LGMD2F) that are caused, respectively, by mutations in the *SGCG*, *SGCA*, *SGCB*, and *SGCD* genes. Knowledge about the clinical and genetic features of sarcoglycanopathies in Chinese patients is limited. The aims of this study were to investigate in detail the clinical manifestations, sarcoglycan expression, and gene mutations in Chinese patients with sarcoglycanopathies and to identify possible correlations between them.

**Results:**

Of 3638 patients for suspected neuromuscular diseases (1733 with inherited myopathies, 1557 with acquired myopathies, and 348 unknown), 756 patients had next-generation sequencing (NGS) diagnostic panel. Twenty-five patients with sarcoglycanopathies (11.5%) were identified from 218 confirmed LGMDs, comprising 18 with LGMD2D, 6 with LGMD2E, and one with LGMD2C. One patient with LGMD2D also had Charcot-Marie-Tooth 1A. The clinical phenotypes of the patients with LGMD2D or LGMD2E were markedly heterogeneous. Muscle biopsy showed a dystrophic pattern in 19 patients and mild myopathic changes in 6. The percentage of correct prediction of genotype based on expression of sarcoglycan was 36.0% (4 LGMD2D, 4 LGMD2E, and one LGMD2C). There was a statistically significant positive correlation between reduction of α-sarcoglycan level and disease severity in LGMD2D. Thirty-five mutations were identified in *SGCA*, *SGCB*, *SGCG*, and *PMP22*, 16 of which were novel. Exon 3 of *SGCA* was a hotspot region for mutations in LGMD2D. The missense mutation c.662G > A (p.R221H) was the most common mutation in *SGCA*. Missense mutations in both alleles of *SGCA* were associated with a relative benign disease course. No obvious clinical, sarcoglycan expression, and genetic correlation was found in LGMD2E.

**Conclusions:**

This study expands the clinical and genetic spectrum of sarcoglycanopathies in Chinese patients and provides evidence that disease severity of LGMD2D may be predicted by α-sarcoglycan expression and *SGCA* mutation.

**Electronic supplementary material:**

The online version of this article (10.1186/s13023-019-1021-9) contains supplementary material, which is available to authorized users.

## Background

Sarcoglycanopathies comprise four subtypes of autosomal recessive limb-girdle muscular dystrophy (LGMD2C, LGMD2D, LGMD2E, and LGMD2F) that are caused, respectively, by mutations in the *SGCG*, *SGCA*, *SGCB*, and *SGCD* genes, which encode four transmembrane glycoproteins, i.e., γ-sarcoglycan (SG), α-SG, β-SG, and δ-SG [1]. The subtypes of sarcoglycanopathy vary in prevalence according to ethnicity and geographic region. LGMD2D is relatively common in Europe and the US [2–4], whereas LGMD2E is most common in the Iranian population [5] and LGMD2C is most common in the Indian [6] and Algeria [7] populations. The typical clinical phenotype of a sarcoglycanopathy includes progressive muscle weakness and atrophy, predominantly of the shoulder and pelvic girdles, and elevated serum creatine kinase (CK). However, there is marked heterogeneity in the clinical phenotype, which ranges from a severe Duchenne-like muscular dystrophy to a mild form that manifests as asymptomatic hyperCKemia or exercise-induced myalgia and myoglobinuria [2, 4, 8–11]. Moreover, it has been reported that reduced or absent sarcolemmal expression of one or all of the four sarcoglycans (SGs) can be found in patients with LGMD2C-F, suggesting that residual expression of sarcoglycan does not accurately predict the genotype in a patient with sarcoglycanopathy [12]. Therefore, accurate diagnosis of sarcoglycanopathy relies mainly on genetic analysis.

To the best of our knowledge, there are only three published investigations of Chinese patients with sarcoglycanopathies [13–15], all of which included very small number of patients and lacked comprehensive genotype-phenotype analysis. Therefore, knowledge about the clinical and genetic features of sarcoglycanopathies in Chinese patients is limited. The aims of this study were to investigate in detail the clinical manifestations, SG expression, and gene mutations in a Chinese population with sarcoglycanopathies and to identify possible correlations between phenotype, genotype, and SG expression.

## Materials and methods

### Patients

Of 3638 patients who underwent muscle biopsy for a suspected neuromuscular disorder (1733 with inherited myopathies, 1557 with acquired myopathies, and 348 unknown) at Peking University First Hospital from January 2013 to August 2018, 756 patients highly suspected of inherited myopathies had next-generation sequencing (NGS) diagnostic panel covering all exons and flanking sequences of genes known to be associated with inherited neuromuscular diseases (Additional file 1: Table S1) according to the following inclusion and exclusion criteria. Inclusion criteria: 1) clinically presented with muscle weakness verified by muscle-strength examination, delayed motor milestones, muscle pain, or exercise intolerance; 2) muscle biopsy showing (1) dystrophic or myopathic changes, i.e., the presence of degenerated and regenerated muscle fibers, with or without variation in fiber size, proliferation of connective tissue, and/or (2) immunohistochemical staining or western blot results showing either decreased expression or accumulation of muscle related proteins; 3) agreed to provide DNA samples for NGS. Exclusion criteria: 1) clinical, histopathologic, and/or genetic diagnosis of facioscapulohumeral muscular dystrophy or myotonic muscular dystrophy; 2) deletion/duplication of exons detected in the *DMD* gene using multiplex ligation-dependent probe amplification (MLPA) assay; 3) muscle biopsy and genetic confirmation of mitochondrial myopathy, glycogen storage myopathy, or lipid storage myopathy; 4) muscle biopsy confirmation of normal histological appearance without any specific pathological findings [13]. Of the 441 patients showing varying reduction of sarcoglycans with or without reduction of dystrophin on muscle biopsy, 25 were confirmed to have the primary genetic defect in *SGCA*, *SGCB*, and *SGCG*, 2 were confirmed to have the primary genetic defect in *FKRP*, and 392 were confirmed to have the primary genetic defect in *DMD*. The primary genetic defect in the remaining 22 patients remained unclear. A total of 218 patients were diagnosed with LGMD based on their clinical manifestations, muscle biopsy results, and genetic analysis, 25 of which were diagnosed with sarcoglycanopathies. Eighteen of these 25 patients were confirmed to have LGMD2D, 6 to have LGMD2E, and one to have LGMD2C, with these patients originating from 12 separate provinces in China (Additional file 2: Figure S1). The proportion of different LGMD subtypes was shown in Additional file 3: Figure S2. Clinical characteristics at the time of diagnosis were evaluated by review of medical records and a detailed physical examination. Walking ability was graded from 1 to 5 according to the scoring system devised by Tasca et al. [4]. Muscle strength was evaluated by manual muscle testing and graded according to Medical Research Council.

### Genetic tests

Genomic DNA was extracted using standard procedures from peripheral blood samples or muscle tissues taken from all patients. Sequence variants were detected by NGS diagnostic panel (Additional file 1: Table S1). Sanger sequencing with specific primers was performed to confirm the variants detected by NGS. In patients who had large deletion or large duplication variants detected by NGS, we further performed MLPA assay (patients 10, 11, and 15) or fluorescence quantitative polymerase chain reaction (patient 19) to confirm these variants. MLPA was also performed in four patients with only one mutation identified in *SGCA* or *SGCB* to rule out deletions/duplications on the other allele. Variants were described according to the Human Genome Variation Society (HGVS) nomenclature using nucleotide and amino acid numbering based on published coding DNA reference sequences (*SGCA*, NM_000023.2; *SGCB*, NM_000232.4; *SGCG*, NM_000231.2; and *PMP22*, NM_000304.2) and protein reference sequences (*SGCA*, NP_000014.1; *SGCB*, NP_000223.1; *SGCG*, NP_000222.1; and *PMP22*, NP_000295.1).

### Clinical interpretation of sequence variants detected in this study

When interpreting and classifying a sequence variant in our study population, we checked to see if it has been previously reported as a pathogenic variant in the Human Gene Mutation Database [16], ClinVar [17], and Google Scholar [18]. Each novel sequence variant was classified as pathogenic, likely pathogenic, uncertain significance, likely benign, or benign according to the rules specified in the 2015 American College of Medical Genetics and Genomics and Association for Molecular Pathology (ACMG-AMP) guidelines [19].

When assessing the frequencies of variants in large populations, 100 healthy control participants (100HC) of Chinese origin were screened, and we also checked for allele frequencies in the Genome Aggregation Database (gnomAD) [20], NHLBI Exome Sequencing Project (ESP6500) Exome Variant Server [21], 1000 Genomes Project (TGP) [22], and Exome Aggregation Consortium (ExAC) [23]. The evidence for pathogenicity was deemed to be moderate (PM2) for variants that were absent or present at extremely low frequencies with alternative allele frequency < 0.5% [24] in population databases. Multiple pieces of computational evidence were derived from various in silico analyses where the FATHMM [25], Mutation Taster [26], PolyPhen-2 [27], and SIFT [28] were used to predict deleteriousness and GERP [29] was used to assess evolutionary conservation. The splicing impact of a variant spanning the exon and intron region was inferred by the Human Splicing Finder (HSF) [30]. Segregation analysis of the variants was performed in available family members. We used the wInterVar tool [24] to automatically generate predictions on 6 (PS1, PM1, PM5, PP2, BP1, BP7) of 28 criteria specified in the 2015 ACMG-AMP guidelines; the rest were interpreted by manual review and adjustment on the basis of variants’ detailed information (such as a variant’s de novo status) and our own domain knowledge. These criteria were then combined to arrive at a final interpretation.

### Muscle biopsy and immunohistochemistry

The muscle biopsies were evaluated and rated by two independent evaluators (WZ and YY), both of whom were experienced in interpretation of muscle biopsies and muscle immunoanalysis and blinded to the underlying genotypes of the patients. Muscle biopsies were obtained from quadriceps femoris (patients 6 and 8), gastrocnemius (patients 4 and 11), tibialis anterior (patients 10, 16 and 20), or biceps brachii (patients 1–3, 5, 7, 9, 12–15, 17–19, and 21–25, and the normal control subjects). The muscle specimens were frozen in isopentane, cooled in liquid nitrogen, and then stored at − 80 °C. Routine histological and histochemical staining was performed [31] and standard techniques were used for immunohistochemical staining [32]. Primary antibodies against the following proteins were used: α-SG, β-SG, and γ-SG (all from Leica Biosystems Newcastle Ltd., Newcastle upon Tyne, UK). Protein expression on sections was scored according to the intensity of staining of the sarcolemma as follows [12]: score 1, normal (complete staining of all fibers); score 2, slight reduction (partial or incomplete staining of a few fibers); score 3, reduction (between severe reduction and slight reduction); score 4, severe reduction (partial or incomplete staining of most fibers); score 5, absence (absence of staining of cell membrane). The genotype was predicted on the basis of the rule that the SG (α, β, or γ) with the most severely reduced expression was the one primarily affected; if there was a similar reduction in two or three of the SGs, prediction was considered impossible.

### Statistical analysis

The Shapiro-Wilk test was used to confirm that the measured variables were not normally distributed. The median patient age, age at onset, disease duration, and muscle strength were treated as descriptive statistics. Hierarchical analysis and graphical representation of the muscle strength values in the form of a heatmap were performed using R software version 3.1.3 (The R Foundation for Statistical Computing, Vienna, Austria; http://www.r-project.org). The software established the order of the patients and muscle strength in the heatmap automatically and generated dendrograms that linked patients or muscles with similar involvement. Mann-Whitney U tests were used to compare the main clinical characteristics (age at onset, disease duration, CK value, and disease severity) between patients with LGMD2D and those with LGMD2E. A two-tailed Pearson correlation coefficient (r) was used to analyze the relationship between the main clinical characteristics and the degree of SG protein deficiency. Positive and negative Pearson’s correlations were considered statistically significant if the *P*-value was < 0.01. The statistical analyses were performed using SPSS for Windows version 22.0 (IBM Corp., Armonk, NY, USA).

## Results

### Clinical phenotype

**Fig. 1:**
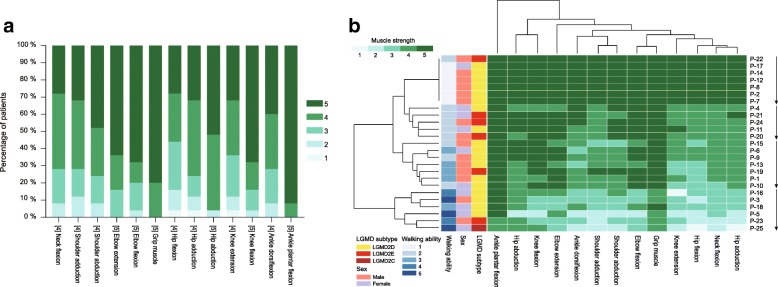
Summary of muscle involvement in patients with sarcoglycanopathies. **a** Green bars indicate the percentage of muscle strength in each muscle group affected with each specified score. The numbers in square brackets represent the median score for each muscle group. **b** A heatmap showing hierarchical clustering of patients and muscle strength according to the scores given to the single muscle groups. Patients do not cluster according to their molecular diagnosis but rather to the severity of muscle involvement. *LGMD* limb-girdle muscular dystrophies

The clinical details of the patients with sarcoglycanopathies were listed in Table 1. Muscle involvement and disease severity determined by hierarchical analysis were shown in Fig. 1. Patients with LGMD2D or LGMD2E did not cluster according to their molecular diagnosis but rather to the severity of muscle involvement. The patients were divided into four subgroups according to the results of the hierarchical analysis, i.e., hyperCKemia without muscle weakness (*n* = 7) and hyperCKemia with muscle weakness that was mild (*n* = 5), intermediate (*n* = 7), or severe (*n* = 6). There was no significant difference in age at onset, disease duration, CK value, or disease severity between the patients with LGMD2D and those with LGMD2E (*P* = 0.545, 0.739, 0.386, and 0.836, respectively). Therefore, the clinical characteristics of the patients with LGMD2D and LGMD2E were summarized together.

**Table 1:** Clinical features in patients with sarcoglycanopathies

Patients	LGMD subtype	Age, years/sex	Age at onset, years	Disease duration, years	Symptom(s) at onset	CK (IU/L)	Walking ability^a^	Distribution of weakness	Motor signs and symptoms				Disease severity^b^
									Calf hypertrophy	Tendon contractures	Scapular winging	Muscle pain	
P1	LGMD2D + CMT1A	9.9/M	2	7.9	delayed motor milestones	18,559	3	Proximal	+	+	–	–	3
P2	LGMD2D	8.8/M	6	2.8	post-exercise muscle pain	8000	1	None	+	–	–	+	1
P3	LGMD2D	16.2/M	2	14.2	frequent falls	345	5	Generalized	+	+	–	–	4
P4	LGMD2D	9.2/F	5	4.2	difficulties in running	10,300	2	Proximal	+	–	–	–	2
P5	LGMD2D	27.4/F	11	16.4	proximal lower limb weakness	763	5	Generalized	–	–	–	–	4
P6	LGMD2D	10.3/F	2	8.3	exercise intolerance	17,690	3	Proximal	+	+	–	–	3
P7	LGMD2D	4.6/M	3.9	0.7	asymptomatic hyperCKemia	6069	1	None	–	–	–	–	1
P8	LGMD2D	13.5/M	10.5	3	post-exercise muscle pain	1770.7	1	None	–	–	–	+	1
P9	LGMD2D	7.4/M	2	5.4	difficulties in running and jumping	8000	2	Proximal	–	–	–	–	3
P10	LGMD2D	11.8/F	9.8	2	difficulties in running and climbing stairs	5355	2	Proximal	–	–	+	–	3
P11	LGMD2D	7.2/F	2.2	5	difficulties in jumping and climbing stairs	12,400	2	Proximal	–	–	–	–	2
P12	LGMD2D	7.7/M	3.7	4	post-exercise muscle pain	8560	1	None	+	–	–	+	1
P13	LGMD2D	9.6/M	7	2.6	proximal lower limb weakness	13,814	3	Proximal	+	+	–	–	3
P14	LGMD2D	8.8/M	2	6.8	post-exercise muscle pain	2650	1	None	+	–	–	+	1
P15	LGMD2D	9/M	1	8	difficulties in climbing stairs	10,347	2	Proximal	–	–	–	+	3
P16	LGMD2D	23.5/F	11	12.5	difficulties in running and climbing stairs	2184	4	Generalized	–	–	+	–	4
P17	LGMD2D	25.5/F	10	15.5	post-exercise muscle pain	400	1	None	–	–	–	+	1
P18	LGMD2D	12.2/F	8.2	4	abnormal gait	3950	3	Generalized	+	+	+	–	4
P19	LGMD2E	12/M	3	9	early fatigue	8022	3	Proximal	+	+	–	–	3
P20	LGMD2E	14.4/M	9.5	4.9	myalgias and exercise intolerance	10,000	2	Proximal	+	+	–	+	2
P21	LGMD2E	9.1/F	8	1.1	proximal lower limb weakness	7873	2	Proximal	–	–	–	–	2
P22	LGMD2E	3.2/M	0.8	2.4	asymptomatic hyperCKemia	35,120	2	None	+	–	–	–	1
P23	LGMD2E	12.9/M	2	10.9	delayed motor milestones	2247	4	Generalized	–	–	–	–	4
P24	LGMD2E	10.7/M	5	5.7	abnormal gait	10,085	2	Proximal	+	+	–	–	2
P25	LGMD2C	29/F	7.5	21.5	frequent falls	842	5	Generalized	–	+	+	–	4

^a^, Walking ability was scored as follows [4]: 1, asymptomatic hyperCKemia with or without exercise-induced myalgia; 2, running with difficulties; 3, unable to run; 4, ambulant with support; 5, non-ambulant. ^b^, Disease severity was determined by hierarchical analysis (Fig. 1b): 1, hyperCKemia without muscle weakness; 2, hyperCKemia with mild muscle weakness; 3, hyperCKemia with intermediate muscle weakness; 4, hyperCKemia with severe muscle weakness. LGMD, limb-girdle muscular dystrophy; CMT1A, Charcot-Marie-Tooth 1A; F, female; M, male; CK, creatine kinase

The median patient age was 10.1 (3.2–27.4) years, the median age at onset was 4.5 (0.8–11) years, and the median duration of disease at the time of diagnosis was 4.6 (0.7–16.4) years. In 16 patients (66.7%), the symptoms at the time of disease onset were associated with proximal lower limb weakness and included early fatigue, frequent falls, gait abnormalities, delayed motor milestones, exercise intolerance, and difficulty in running, climbing and jumping; in 6 patients (25.0%), the symptom at onset was post-exercise muscle pain without muscle weakness. Two patients (8.3%) were diagnosed to have sarcoglycanopathy after an incidental finding of hyperCKemia. Four patients were no longer able to ambulate independently at a median age of 18.2 (range 12–26.4) years. Motor signs included calf hypertrophy (in 54.2% of patients), tendon contractures (in 33.3%), and scapular winging (in 12.5%). Muscle pain was reported by 29.2% of patients. Physical examination revealed that 17 patients (70.8%) had proximal weakness involving the axial, pelvic, and shoulder girdle muscles, and that 7 patients (29.2%) had asymptomatic hyperCKemia or exercise-induced myalgia without muscle weakness. The distal muscles were affected in 5 patients (20.8%), all of whom had a severe disease severity. The hip and neck flexors and hip adductors were the muscle groups most often involved and the plantar flexors were the least often involved.

The patient with LGMD2C in this study (patient 25) had a severe disease severity and was no longer able to ambulate independently by the age of 18 years, and was diagnosed to have sarcoglycanopathy because of frequent falls. In this patient, physical examination revealed proximal and distal muscle weakness as well as tendon contractures and scapular winging.

CK levels were elevated in all patients (345–35,120 IU/L, normal range 25–195 IU/L). Nerve conduction study of patient 1 revealed that the motor nerve conduction velocity (MNCV) and sensory nerve conduction velocity (SNCV) reduced severely in all nerves examined, and the compound muscle action potential (CMAP) amplitude and sensory nerve action potential (SNAP) amplitude decreased in some of the nerves examined (Additional file 4: Table S2). Concomitant mutations in *SGCA* and *PMP22* were confirmed by genetic analysis in patient 1; in this patient, the diagnosis was coexistence of LGMD2D and Charcot-Marie-Tooth 1A (CMT1A).

### Mutations identified in this study

**Fig. 2:**
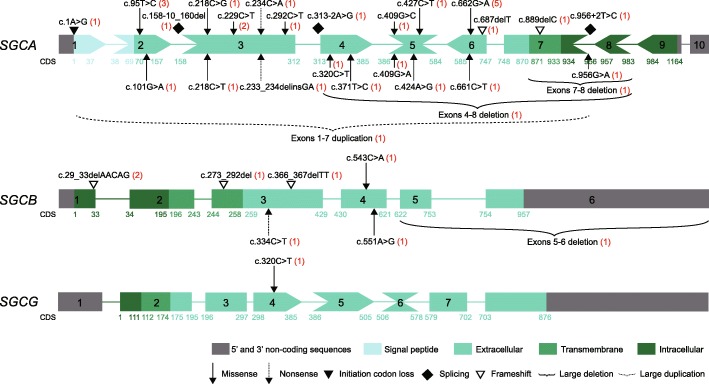
Spectrum and location of mutations in *SGCA*, *SGCB*, and *SGCG*. *SGCA*, *SGCB*, and *SGCG* were represented by their exons. To accommodate the distribution of mutations, the size of the exons was not represented at scale. To illustrate the reading frame, the exons are schematically represented by boxes with blunt, protrusive or intrusive ends. Nucleotide numbering for all mutations was designated according to the coding DNA reference sequence (CDS) in GenBank Accession number NM_000023.2 (*SGCA*), NM_000232.4 (*SGCB*), and NM_000231.2(*SGCG*). Information for the different protein domains is available in https://www.uniprot.org/. Numerals within parentheses indicate, for each mutation, the number of patients harboring the mutation. c.158-10_160del, c.158-10_160delCTTCCACCAGCTG; c.273_292del, c.273_292delCATTGGACCAAATGGCTGTG

In total, 35 mutations were identified in *SGCA* (*n* = 26), *SGCB* (*n* = 7), *SGCG* (*n* = 1) and *PMP22* (*n* = 1), 19 of which have been previously reported as pathogenic [2, 13, 33–42] and the remaining 16 were novel (Table 3). Twenty-one patients (16 with LGMD2D, 4 with LGMD2E, and one with LGMD2C) had a complete molecular diagnosis and were found to have two mutations in *SGCA*, *SGCB* or *SGCG* and 4 (2 with LGMD2D and 2 with LGMD2E) were found to have only one mutation in *SGCA* or *SGCB*. In patient 1, we also identified a previously reported mutation in *PMP22* (duplication of exons 1–5) [41, 42] in addition to the mutations identified in *SGCA*. The allele frequencies in various population databases, results of in silico analysis and clinical interpretation of novel candidate variants detected in *SGCA*, *SGCB* and *SGCG* according to the 2015 ACMG-AMP guidelines were summarized in Tables 2 and 3. Except for the missense variant c.218C > T in *SGCA* (0.00000815 in gnomAD) and the missense variant c.320C > T in *SGCG* (0.00000813 in gnomAD, 0.000008 in ExAC), none of the novel candidate variants were detected in the various population databases or in the 100 healthy control subjects. Not all of the in silico programs tested agreed on the prediction of the missense variant c.956G > A in *SGCA*, so PP3 evidence was not used when classifying this variant. When interpreting and classifying other novel candidate variants that could be predicted by the in silico programs, PP3 evidence was counted as supporting because all of the in silico programs tested agreed on the prediction. After combining the criteria specified in the 2015 ACMG-AMP guidelines [19], all 14 novel candidate variants were classified as pathogenic or likely pathogenic. Spectrum and location of mutations in *SGCA*, *SGCB* and *SGCG* were shown in Fig. 2.

**Table 2:** Allele frequencies in various population databases and in silico analysis of novel candidate variants detected in S*GCA*, *SGCB*, and *SGCG*

Gene	c.DNA position	Global AF in all subpopulation					FATHMM	Mutation Taster	PolyPhen-2 HumVar	SIFT	GERP*
		gnomAD	ESP6500	TGP	ExAC	100 HC					
*SGCA*	c.218C > G	absent	absent	absent	absent	absent	Deleterious	Disease_causing	Probably damaging	Deleterious	4.53
*SGCA*	c.218C > T	0.00000815	absent	absent	absent	absent	Deleterious	Disease_causing	Probably damaging	Deleterious	4.53
*SGCA*	c.158-10_160del	absent	absent	absent	absent	absent	–	–	–	–	–
*SGCA*	c.234C > A	absent	absent	absent	absent	absent	Deleterious	–	–	–	−1.04
*SGCA*	c.427C > T	absent	absent	absent	absent	absent	Deleterious	Disease_causing	Possibly damaging	Tolerated	4.35
*SGCA*	c.956G > A	absent	absent	absent	absent	absent	Deleterious	Disease_causing	Benign	Tolerated	1.19
*SGCA*	c.1A > G	absent	absent	absent	absent	absent	Deleterious	Disease_causing	Possibly damaging	Deleterious	3.78
*SGCA*	c.687delT	absent	absent	absent	absent	absent	–	–	–	–	–
*SGCA*	Exons 1–7 duplication	–	–	–	–	–	–	–	–	–	–
*SGCA*	Exons 4–8 deletion	–	–	–	–	–	–	–	–	–	–
*SGCB*	Exons 5–6 deletion	–	–	–	–	–	–	–	–	–	–
*SGCB*	c.29_33delAACAG	absent	absent	absent	absent	absent	–	–	–	–	–
*SGCB*	c.273_292del	absent	absent	absent	absent	absent	–	–	–	–	–
*SGCB*	c.366_367delTT	absent	absent	absent	absent	absent	–	–	–	–	–
*SGCB*	c.543C > A	absent	absent	absent	absent	absent	Deleterious	Disease_causing	Probably damaging	Deleterious	3.3
*SGCG*	c.320C > T	0.00000813	absent	absent	0.000008	absent	Deleterious	Disease_causing	Probably damaging	Deleterious	5.39

c.158-10_160del, c.158-10_160delCTTCCACCAGCTG; c.273_292del, c.273_292delCATTGGACCAAATGGCTGTG; *, The cutoff was set to 2.0 for GERP (smaller scores indicating less conservation) [24]. AF, allele frequency; gnomAD, Genome Aggregation Database; ESP6500, NHLBI Exome Sequencing Project (ESP6500) Exome Variant Server; TGP, 1000 Genomes Project; ExAC, Exome Aggregation Consortium Browser; 100 HC, 100 healthy control subjects of Chinese origin

**Table 3:** Summary of genetic data and clinical interpretation of novel candidate variants detected in *SGCA*, *SGCB*, and *SGCG* according to the 2015 ACMG-AMP guidelines [19]

Patients	Gene	c.DNA position	Exon	Effect on protein	Type of variants	Location of mutation allele	Parental derivation	Variants pathogenicity	Evidence of pathogenicity
P1	*SGCA*	c.101G > A^a^	Exon 2	p.R34H	Missense	Extracellular	Maternal	Pathogenic	
	*SGCA*	c.218C > G^b^	Exon 3	p.P73R	Missense	Extracellular	Paternal	Likely pathogenic	PM1, PM2, PM3, PP1, PP3, PP4
	*PMP22*	Exons 1–5 duplication^a^	Exons 1–5	–	Large duplication	–	NA	Pathogenic	
P2	*SGCA*	c.158-10_160del^b^	Intron 2, Exon 3	–	Splicing#	Extracellular	Maternal	Likely pathogenic	PM2, PM3, PP1, PP3, PP4
	*SGCA*	c.662G > A^a^	Exon 6	p.R221H	Missense	Extracellular	Paternal	Pathogenic	
P3	*SGCA*	c.662G > A^a^	Exon 6	p.R221H	Missense	Extracellular	NA	Pathogenic	
P4	*SGCA*	c.313-2A > G^a^	Intron 3	NA	Splicing	Extracellular	Maternal	Pathogenic	
	*SGCA*	c.95 T > C^a^	Exon 2	p.V32A	Missense	Extracellular	Paternal	Pathogenic	
P5	*SGCA*	c.424A > G^a^	Exon 5	p.S142G	Missense	Extracellular	NA	Pathogenic	
P6	*SGCA*	c.889delC^a^	Exon 7	p.L298Cfs*23	Frameshift	TM	Maternal	Pathogenic	
	*SGCA*	c.292C > T^a^	Exon 3	p.R98C	Missense	Extracellular	Paternal	Pathogenic	
P7	*SGCA*	c.409G > C^a^	Exon 5	p.E137Q	Missense	Extracellular	Maternal	Pathogenic	
	*SGCA*	c.95 T > C^a^	Exon 2	p.V32A	Missense	Extracellular	Paternal	Pathogenic	
P8	*SGCA*	c.661C > T^a^	Exon 6	p.R221C	Missense	Extracellular	Maternal	Pathogenic	
	*SGCA*	c.320C > T^a^	Exon 4	p.A107V	Missense	Extracellular	Paternal	Pathogenic	
P9	*SGCA*	c.233_234delinsGA^a^	Exon 3	p.Y78*	Nonsense	Extracellular	Maternal	Pathogenic	
	*SGCA*	c.371 T > C^a^	Exon 4	p.I124T	Missense	Extracellular	Paternal	Pathogenic	
P10	*SGCA*	Exons 1–7 duplication^b^	Exons 1–7	–	Large duplication	Singal peptide + Extracellular + TM + Intracellular	Maternal	Pathogenic	PVS1, PM3, PP1, PP4
	*SGCA*	c.229C > T^a^	Exon 3	p.R77C	Missense	Extracellular	Paternal	Pathogenic	
P11	*SGCA*	Exons 4–8 deletion^b^	Exons 4–8	–	Large deletion	Extracellular + TM + Intracellular	Maternal	Pathogenic	PVS1, PM3, PP1, PP4
	*SGCA*	c.234C > A^b^	Exon 3	p.Y78*	Nonsense	Extracellular	Paternal	Pathogenic	PVS1, PM2, PP4
P12	*SGCA*	c.956 + 2 T > C^a^	Intron 7	–	Splicing	Intracellular	Maternal	Pathogenic	
	*SGCA*	c.662G > A^a^	Exon 6	p.R221H	Missense	Extracellular	Paternal	Pathogenic	
P13	*SGCA*	c.427C > T^b^	Exon 5	p.H143Y	Missense	Extracellular	Maternal	Likely pathogenic	PM2, PM3, PP1, PP3, PP4
	*SGCA*	c.229C > T^a^	Exon 3	p.R77C	Missense	Extracellular	Paternal	Pathogenic	
P14	*SGCA*	c.662G > A^a^	Exon 6	p.R221H	Missense	Extracellular	Maternal	Pathogenic	
	*SGCA*	c.956G > A^b^	Exon 7	p.R319K	Missense	Intracellular	Paternal	Likely pathogenic	PM2, PM3, PP1, PP4
P15	*SGCA*	Exons 7–8 deletion^a^	Exons 7–8	–	Large deletion	TM + Intracellular	Maternal	Pathogenic	
	*SGCA*	c.218C > T^b^	Exon 3	p.P73L	Missense	Extracellular	Paternal	Likely pathogenic	PM1, PM2, PM3, PP1, PP3, PP4
P16	*SGCA*	c.1A > G^b^ (hom)	Exon 1	p.0?	Init-loss	Singal peptide	Maternal/Paternal	Pathogenic	PVS1, PM2, PP1, PP3, PP4
P17	*SGCA*	c.662G > A^a^	Exon 6	p.R221H	Missense	Extracellular	Maternal	Pathogenic	
	*SGCA*	c.95 T > C^a^	Exon 2	p.V32A	Missense	Extracellular	Paternal	Pathogenic	
P18	*SGCA*	c.409G > A^a^	Exon 5	p.E137K	Missense	Extracellular	Maternal	Pathogenic	
	*SGCA*	c.687delT^b^	Exon 6	p.L230Cfs*18	Frameshift	Extracellular	Paternal	Pathogenic	PVS1, PM2, PM3, PP1, PP4
P19	*SGCB*	c.551A > G^a^	Exon 4	p.Y184C	Missense	Extracellular	Maternal	Pathogenic	
	*SGCB*	Exons 5–6 deletion^b^	Exons 5–6	–	Large deletion	Extracellular	Paternal	Pathogenic	PVS1, PM3, PP1, PP4
P20	*SGCB*	c.334C > T^a^	Exon 3	p.Q112*	Nonsense	Extracellular	Paternal	Pathogenic	
P21	*SGCB*	c.29_33delAACAG^b^ (hom)	Exon 1	p.E10Afs*13	Frameshift	Intracellular	Maternal/Paternal	Pathogenic	PVS1, PM2, PP1, PP4
P22	*SGCB*	c.273_292del^b^ (hom)	Exon 3	p.I92*	Frameshift	TM	NA	Pathogenic	PVS1, PM2, PP4
P23	*SGCB*	c.29_33delAACAG^b^	Exon 1	p.E10Afs*13	Frameshift	Intracellular	Maternal	Pathogenic	PVS1, PM2, PP1, PP4
	*SGCB*	c.366_367delTT^b^	Exon 3	p.Y123*	Frameshift	Extracellular	Paternal	Pathogenic	PVS1, PM2, PP1, PP4
P24	*SGCB*	c.543C > A^b^	Exon 4	p.S181R	Missense	Extracellular	Maternal	Likely pathogenic	PS1, PM2, PP3, PP4
P25	*SGCG*	c.320C > T^b^ (hom)	Exon 4	p.S107 L	Missense	Extracellular	Maternal/Paternal	Likely pathogenic	PM2, PM3, PP1, PP3, PP4

^a^, The variants have been previously reported as pathogenic [2, 13, 33–42]; ^b^, novel variants; #, This mutation most likely affects splicing because it can cause loss of the acceptor splice sites, as confirmed by HSF Matrices and MaxEnt algorithms [30]. Init-loss, initiation codon loss; hom, homozygous; *NA* not available, *TM* transmembrane, *PVS* pathogenic very strong, *PS* pathogenic strong, *PM* pathogenic moderate, *PP* pathogenic supporting; c.158-10_160del, c.158-10_160delCTTCCACCAGCTG; c.273_292del, c.273_292delCATTGGACCAAATGGCTGTG

#### *SGCA*

Twenty-six mutations were identified in *SGCA*, 16 of which have been reported previously (Table 3), including 11 missense mutations, 2 splicing mutations, one nonsense mutation, one small deletion, and one large deletion. The remaining ten mutations were novel and included 4 missense mutations (c.218C > T (p.P73L), c.218C > G (p.P73R), c.427C > T (p.H143Y), and c.956G > A (p.R319K)), one nonsense mutation (c.234C > A (p.Y78*)), one mutation (c.1A > G) that caused loss of the initiation codon, one splicing mutation (c.158-10_160delCTTCCACCAGCTG), one small deletion (c.687delT (p.L230Cfs*18)), one large deletion (a deletion of exons 4–8), and one large duplication (a duplication of exons 1–7). The initiation codon loss mutation c.1A > G was a homozygous state in patient 16. Except for the mutations found in 2 patients with only one mutation, other mutations were the compound heterozygous state in patients with LGMD2D.

Fifteen of the 26 mutations were missense mutations that accounted for 64.7% of the mutated alleles. Seven (26.9%) of 26 mutations were located in exon 3. All 15 missense mutations but one (c.956G > A in the intracellular domain) affected amino acids located in the extracellular domain of α-SG (Fig. 2). Three missense mutations were found to be recurrent and accounted for 29.4% of the mutated alleles. The missense mutation c.662G > A in *SGCA*, carried by 5 unrelated patients (27.8%) from different geographic regions but relatively concentrated in East China (Additional file 2: Figure S1) and accounting for 14.7% of the mutated alleles, was the most common mutation in the patients with LGMD2D. The missense mutation c.95 T > C was identified in 3 patients with compound heterozygosity (16.7%) and of diverse geographic origin (Additional file 2: Figure S1), and the missense mutation c.229C > T was found in 2 patients (11.1%).

#### *SGCB*

We identified a total of 7 mutations in *SGCB*, which included the previously reported missense mutation c.551A > G (p.Y184C) [40] and nonsense mutation c.334C > T (p.Q112*) [13] (Table 3). The 5 novel mutations included 3 small deletions (c.29_33delAACAG (p.E10Afs*13), c.273_292delCATTGGACCAAATGGCTGTG (p.I92*), and c.366_367delTT (p.Y123*)), one missense mutation (c.543C > A (p.S181R)), and one large deletion (a deletion of exons 5–6). Null mutations (nonsense mutation, small deletions, and large deletions) accounted for 80% of the mutated alleles. The deletion of exons 5–6 that could result in a truncated β-SG with 112 amino acids less than normal protein, and the missense mutation c.551A > G in *SGCB* were observed to be in the compound heterozygous state in patient 19. The mutations c.29_33delAACAG and c.273_292delCATTGGACCAAATGGCTGTG were observed in the homozygous state in patients 21 and 22, respectively. Compound heterozygous mutations c.29_33delAACAG and c.366_367delTT were observed in patient 23. Only one mutation was identified in patients 20 and 24.

#### *SGCG*

The homozygous missense mutation c.320C > T in *SGCG* identified in one patient with LGMD2C was novel mutation.

### Muscle biopsy and immunohistochemistry

**Fig. 3:**
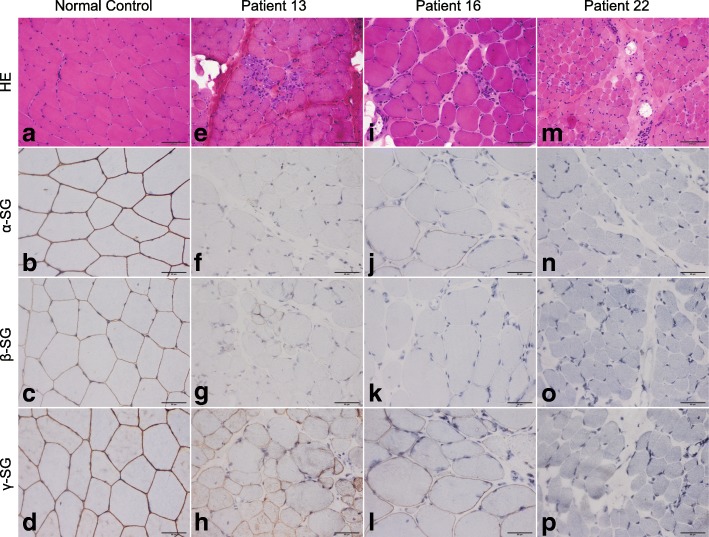
Pathologic changes and immunohistochemistry analysis of sarcoglycans in patients with sarcoglycanopathies. **a** HE staining showing no pathologic changes; (**e**, **i**, **m**) HE staining showing a dystrophic pattern in patients 13, 16, and 22; (**b**–**d**) a normal control subject showing positive staining of the three sarcoglycans (score 1); (**f**–**h**) a representative case of LGMD2D showing severe reduction of α-SG and β-SG expression (score 4) and slight reduction of γ-SG expression (score 2); (**j**–**l**) a representative case of LGMD2D showing severe reduction of α-SG expression (score 4), no expression of β-SG (score 5), and reduction of γ-SG expression (score 3); and (**n**–**p**) a representative case of LGMD2E showing no expression of any of the three sarcoglycans (score 5). HE, Hematoxylin-eosin staining (200× magnification); SG, sarcoglycan (400× magnification)

The results of muscle biopsy and immunohistochemistry analysis were summarized in Fig. 3 and Additional file 5: Table S3. The majority of muscle biopsy specimens (76.0%) showed a dystrophic pattern, i.e., increased variation in fiber size, proliferation of connective tissue, and necrotic and regenerated fibers. The muscle biopsies in 6 patients with LGMD2D who had a mild form of disease severity showed mild myopathic changes, including a few hypertrophic, atrophic, hypercontracted and whorled fibers, as well as fiber splitting and a small number of internal nuclei. We were able to correctly predict the genotype in 36.0% of the patients according to α-, β-, or γ-SG that was most reduced on the sections. In 52.0% patients, it was impossible to predict the genotype because there was a similar reduction in expression of two or three of α-, β-, and γ-SG. Furthermore, the prediction was incorrect in 12.0% of patients.

In the patients with LGMD2D, there was variable reduction in expression of α-SG, ranging from a slight decrease to absence, except in patient 17, in whom expression of α-SG was positive and that of β-SG was slightly reduced. Expression of β-SG and γ-SG was also affected, with varying degrees of deficiency in patients with LGMD2D, except for 3 patients. In 14 patients, expression of α-SG was found to be similarly or more severely reduced than the expression levels of β-SG and/or γ-SG; in 3 patients, the most pronounced reduction was in β-SG. In the patients with LGMD2E, β-SG was absent in all but one case (patient 19), and expression levels of α-SG and γ-SG were reduced to varying degrees. The expression of β-SG was found to be more decreased than that of α-SG and γ-SG in 4 patients and reduced to a similar extent of α- and/or γ-SG in 2 patients. There was a concomitant absence of α-SG, β-SG, and γ-SG in one patient with LGMD2D (patient 1) and in one with LGMD2E (patient 22). In the patient with LGMD2C, the expression levels of all three SGs were decreased, particularly for γ-SG.

### Correlation of phenotype, genotype, and SG expression levels

No statistically significant correlations were found between age at onset, duration of disease, CK value, and disease severity in the patients with LGMD2D or LGMD2E. There was a statistically significant positive correlation of reduction of the α-SG level with disease severity in the patients with LGMD2D (*r* = 0.689, *P* = 0.002), indicating that the greater the amount of residual protein, the milder the disease. This correlation was not found in patients with LGMD2E.

Six (60.0%) of 10 patients with LGMD2D who harbored null mutations (splicing, nonsense, initiation codon loss, large deletion or duplication, and frameshift mutations) in at least one of the mutated alleles had severe forms of disease severity (hyperCKemia with intermediate or severe muscle weakness) and the remaining 4 (40.0%) had mild forms (hyperCKemia without muscle weakness or hyperCKemia with mild muscle weakness). Immunohistochemistry analysis showed a similar pattern of reduction, i.e., a marked decrease in or absence of α-SG with variable reduction in β-BG and/or γ-SG. The only exception was patient 12, who harbored a canonical splicing mutation c.956 + 2 T > C in *SGCA* and showed a slight reduction in α-SG on immunohistochemical staining. In 6 LGMD2D patients with missense mutations in both alleles, 4 (66.7%) of them had mild forms of disease severity and showed a slight reduction in α-SG or positive staining but with a slight reduction in β-SG (patient 17), while the other 2 patients had a severe form and a drastic decrease or absence of α-SG. Four (80%) of the 5 patients carrying the missense mutation c.662G > A in one allele had hyperCKemia without muscle weakness, regardless of the null mutation in the other allele in two of them.

Although null mutations were identified in at least one allele in 5 patients with LGMD2E, their disease severity varied from mild forms to severe forms. However, all the muscle biopsies in these patients showed a marked decrease or absence of β-SG. In patient 24 who had the missense mutation c.543C > A in *SGCB* and a mild disease severity, the expression of β-SG was absent. No obvious genotype-phenotype correlation was found in the patients with LGMD2E.

## Discussion

In this study, we undertook a comprehensive analysis of the clinical phenotypes, SG expression, and genetic data in 25 unrelated patients with sarcoglycanopathies who originated from 12 separate provinces in mainland china. These 25 patients were identified among 3638 patients in whom neuromuscular diseases were suspected. Most of the patients (88.9%) showing varying reduction of sarcoglycans with or without reduction of dystrophin were confirmed to have the primary genetic defect in *DMD* and showed varying reduction of dystrophin, mainly because the dystrophinopathy is most common among different muscular dystrophies related to the dystrophin-glycoprotein complex and a primary defect in a protein of the complex can cause a secondary reduction of other proteins of the complex [12], which indicating that the *DMD* gene should be firstly and cautiously analyzed in a patient showing varying reduction of sarcoglycans and dystrophin. We believe this to be the largest series of Chinese patients with sarcoglycanopathies investigated to date. Eighteen (72.0%) of these patients had the LGMD2D subtype, 6 (24.0%) had LGMD2E, one (4.0%) had LGMD2C, and none had LGMD2F. Therefore, it seems probable that the LGMD2C and LGMD2F subtypes of sarcoglycanopathies are rarer in China than in other countries [2, 3, 5, 12] and that LGMD2D is more common, as is the case in Taiwan [15]. In our cohort, the second most common type of sarcoglycanopathies was LGMD2E, which is different to the reports of the second common type being LGMD2C in Europe and the US [4] and LGMD2F in Brazil [43] and India [6].

We found that the phenotypic spectrum in patients with LGMD2E was similar to that in those with LGMD2D in terms of age at onset, disease duration, CK value, and disease severity, which is in contrast with several previous reports of the clinical phenotypes in patients with LGMD2D being milder than those in their counterparts with LGMD2E [36, 44–46]. As in other studies [2, 4, 8–12, 47], we also found that the clinical phenotypes in patients with LGMD2D or LGMD2E were heterogeneous and covered a broad clinical spectrum, ranging from a severe Duchenne-like dystrophy to a mild form manifesting as asymptomatic hyperCKemia or hyperCKemia with exercise-induced myalgia but without muscle weakness. Furthermore, in our study, patients with a severe disease severity had distal muscle involvement, which has been described previously [11]. Like the patient with missense mutations in *SGCG* and severe disease described in an earlier report [5], the only patient in our cohort with LGMD2C and homozygous missense mutations in *SGCG* had a severe disease severity. Surprisingly, we identified concomitant mutations in *SGCA* and *PMP22* in one patient, whose nerve conduction study results were compatible with CMT1A. Therefore, we consider that the diagnosis in this patient was coexistence of LGMD2D and CMT1A. To the best of our knowledge, this is the first confirmed case of concomitant LGMD2D and CMT1A reported in the literature. The presence of two distinct genetic forms, i.e., LGMD2D and CMT1A, in the same patient highlights the complexity of genetic counselling in patients with sarcoglycanopathies. Neurologists and pediatricians should be aware of this possibility.

As in previous studies [2, 11, 12, 48], muscle biopsies and immunohistochemistry in our patients revealed a dystrophic pattern and mild myopathic changes with a highly variable pattern of SG expression, which included reduced or absent sarcolemmal expression of one or all of the α-, β- and γ-SG. We were only able to correctly predict the genotype in 36.0% of the patients in whom α-, β-, or γ-SG was most reduced. In 52.0% of our patients, it was impossible to predict the genotype because there was a similar reduction in expression of two or three of α-, β-, and γ-SG. Moreover, the prediction was incorrect in 12.0% of patients resulting from the fact that β-SG was most reduced in 3 patients with LGMD2D. Therefore, muscle immunoanalysis did not accurately predict the primary defect in the majority of these patients, as in a previous report [12]. Some of the patients with LGMD2D who had a mild clinical course showed only mild myopathic changes and mild reduction in α-SG expression, which is consistent with the general understanding that, usually, patients with mild disease have mild pathologic changes, and this has also been observed in a previous study [10]. A statistically significant positive correlation between reduction of α-SG level and disease severity was observed in patients with LGMD2D, indicating that disease severity may be predicted by α-SG expression in these patients. In contrast with a previous report suggesting that disease severity might be predicted by the β-SG expression level in patients with LGMD2E [11], we did not find any relationship between β-SG expression and disease severity in our patients with this subtype.

Genetic analysis of the sarcoglycanopathies is still a difficult task, in that we only identified one mutation in four of our patients with LGMD2D and LGMD2E and found various types of mutations in *SGCA*, *SGCB*, and *SGCG*. Another mutation remains unknown and might be deep intronic variants on the other allele; for example, the homozygous intronic deletion of *SGCA* has been described in a patient with LGMD2D [9]. Seven of 26 mutations was located in exon 3, suggesting that exon 3 is a hotspot region for mutations in *SGCA* in Chinese patients with LGMD2D and that it should be cautiously analyzed. We found that the initiation codon loss mutation c.1A > G was a homozygous base pair substitution in the translation initiation codon of *SGCA*, which was found in one patient with LGMD2D who had severe disease. This mutation is predicted to influence initiation of translation at the mRNA position, possibly decreasing the amount of protein translated from the first AUG codon and allowing recognition of the next methionine codon in the appropriate context, i.e., the Kozak consensus sequence [49], as the start site. The ATGpr algorithm [50] confirmed the hypothesis that the reading frame would be maintained in this specific condition, but the encoded protein would miss the first 211 amino acid residues. The novel variant c.158-10_160delCTTCCACCAGCTG is a splicing mutation that spans exon 3 and intron 2 region of *SGCA*. This mutation most likely affects splicing because it can cause loss of the acceptor splice sites, as confirmed by HSF Matrices and MaxEnt algorithms [30].

Missense mutations were common in our patients with LGMD2D. All but one missense mutation affected amino acids positioned in the extracellular domain of α-SG, which is not unexpected because the extracellular domain of α-SG is very large and is constituted by the vast majority of the amino acids of α-SG. The missense mutation c.662G > A in *SGCA* was the most common mutation found in patients with LGMD2D who originated from East China, whereas the most common mutation is c.101G > T (R34L) in Taiwan [15] and c.229C > T (R77C) in several other countries [2, 46, 51, 52]. Similar to the findings of previous studies [2, 5, 11, 12, 15, 36, 39, 46, 53], the results of our present study suggest that, in contrast with the predominant presence of missense mutations in LGMD2D, null mutations were more prevalent in LGMD2E.

Our study suggests that the disease severity of LGMD2D may be related to the type of mutations. Most of our LGMD2D patients who harbored two missense mutations had mild forms of disease severity, therefore, to some extent, LGMD2D patients with missense mutations in *SGCA* in both alleles might have a mild disease course, as reported by other researchers [8, 54]. However, we could not conclude that null mutations in patients with LGMD2D were associated with a severe disease course because of the variation in disease severity of our patients with LGMD2D and null mutations in *SGCA*. Some studies have found that null mutations in *SGCA* were also associated with mild disease severity [9, 10]. Four of the 5 LGMD2D patients carrying the c.662G > A mutation had hyperCKemia without muscle weakness despite two of them having the null mutation on the other allele, indicating that the missense mutation c.662G > A was associated with a benign disease course. We found no obvious genotype-phenotype correlation in our patients with LGMD2E, whereas a previous study found that disease severity might be predicted by *SGCB* mutation and expression of β-SG [11].

In conclusion, the results of this study illustrate that both muscle biopsy and genetic analysis remain essential methods for the correct diagnosis of sarcoglycanopathies. LGMD2D is the most common type of sarcoglycanopathies in China. We identified 16 novel mutations in *SGCA*, *SGCB*, and *SGCG* in 25 patients, who showed a broad spectrum of clinical phenotypes, and identified for the first time a patient with coexistence of LGMD2D and CMT1A. This study provides evidence that disease severity of LGMD2D may be predicted by α-SG expression and *SGCA* mutation. These findings expand our knowledge of the clinical and genetic spectrum of sarcoglycanopathies in Chinese patients.

## Additional files

Additional file 1: **Table S1.** Four hundred and twenty genes included in the neuromuscular disease panel. (DOCX 18 kb)

Additional file 2: **Figure S1.** Geographic origin of Chinese patients with sarcoglycanopathies and the common mutations identified in *SGCA*. (TIF 3913 kb)

Additional file 3: **Figure S2.** The proportion of different LGMD subtypes. (A) Overview of LGMD subtypes, including autosomal dominant (LGMD1) or autosomal recessive (LGMD2) types. (B) LGMD1 comprised LGMD1E and LGMD1B. (C) The proportion of different LGMD2 subtypes. (TIF 2636 kb)

Additional file 4: **Table S2.** Nerve conduction study of patient 1. (DOCX 15 kb)

Additional file 5: **Table S3.** Pathologic changes and prediction of genotype based on expression of sarcoglycans in patients with sarcoglycanopathies. (DOCX 20 kb)

## Abbreviations

100HC: 100 healthy control participants
ACMG-AMP: American College of Medical Genetics and Genomics and Association for Molecular Pathology
CK: Creatine kinase
CMAP: Compound muscle action potential
CMT1A: Charcot-Marie-Tooth 1A
ESP6500: NHLBI Exome Sequencing Project (ESP6500) Exome Variant Server
ExAC: Exome Aggregation Consortium
gnomAD: Genome Aggregation Database
HGVS: Human Genome Variation Society
HSF: Human splicing finder
LGMD: Limb-girdle muscular dystrophy
MLPA: Multiplex ligation-dependent probe amplification
MNCV: Motor nerve conduction velocity
NGS: Next-generation sequencing
SG: Sarcoglycan
SGs: Sarcoglycans
SNAP: Sensory nerve action potential
SNVC: Sensory nerve conduction velocity
TGP: 1000 Genomes Project
