# Supplementary material for: Clinical and genetic spectrum of sarcoglycanopathies in a large cohort of Chinese patients
Source: Orphanet J Rare Dis. 2019 Feb 14;14:43. doi: 10.1186/s13023-019-1021-9 (PMC6376703; doi:10.1186/s13023-019-1021-9)
Supplement: Supplementary file 1 — Table S1. Four hundred and twenty genes included in the neuromuscular disease panel. (DOCX 18 kb) [file 13023_2019_1021_MOESM1_ESM.docx]

Table S1. Four hundred and twenty genes included in the neuromuscular disease panel.

| *AAAS* | *AARS* | *ABC1* | *ABCA1* | *ABCD1* | *ABHD12* | *ABHD5* |
| --- | --- | --- | --- | --- | --- | --- |
| *ACAD8* | *ACADL* | *ACADM* | *ACADS* | *ACADVL* | *ACTA1* | *ACVR1* |
| *ADCK3* | *AGC1* | *AGL* | *AGRN* | *AIFM1* | *AIRE* | *ALDOA* |
| *ALDOB* | *ALG1* | *ALG12* | *ALG13* | *ALG14* | *ALG2* | *ALG3* |
| *ALG6* | *ALG8* | *ALG9* | *ALS2* | *AMACR* | *ANO5* | *APOA1* |
| *ARHGEF10* | *ARL6IP1* | *ARSA* | *ASAH1* | *ASL* | *ATL1* | *ATL3* |
| *ATM* | *ATP7A* | *ATP7B* | *B3GALNT2* | *B3GNT1* | *B4GALT1* | *BAG3* |
| *BICD2* | *BIN1* | *BSCL2* | *C10orf2* | *C7orf10* | *CACNA1A* | *CACNA1C* |
| *CACNA1S* | *CACNB2* | *CACNL2D1* | *CAPN3* | *CASQ1* | *CAV3* | *CCDC78* |
| *CCT5* | *CFL2* | *CHAT* | *CHCHD10* | *CHKB* | *CHN1* | *CHRM3* |
| *CHRNA1* | *CHRNB* | *CHRNB1* | *CHRND* | *CHRNE* | *CHRNG* | *CLCN1* |
| *CLN3* | *CNTN1* | *COG1* | *COG7* | *COG8* | *COL12A1* | *COL25A1* |
| *COL6A1* | *COL6A2* | *COL6A3* | *COL9A2* | *COL9A3* | *COLQ* | *COMP* |
| *COQ2* | *COQ6* | *COQ9* | *COX6A1* | *CP* | *CPT1A* | *CPT2* |
| *CRAT* | *CRYAB* | *CTDP1* | *CUL4B* | *DAG1* | *DCAF8* | *DCTN1* |
| *DECR1* | *DES* | *DGUOK* | *DHH* | *DHTKD1* | *DMD* | *DNAJB2* |
| *DNAJB6* | *DNAJC3* | *DNM2* | *DNMT1* | *DOK7* | *DOLK* | *DPAGT1* |
| *DPM1* | *DPM2* | *DPM3* | *DST* | *DYNC1H1* | *DYSF* | *ECEL1* |
| *EGR2* | *EMD* | *ENO3* | *EPM2A* | *ETFA* | *ETFB* | *ETFDH* |
| *ETHE1* | *EXOSC3* | *EXOSC8* | *FAM134B* | *FBLN5* | *FBP1* | *FBXO38* |
| *FGA* | *FGD4* | *FHL1* | *FIF1B* | *FIG4* | *FKBP14* | *FKRP* |
| *FKTN* | *FLNA* | *FLNC* | *FLVCR1* | *FUS* | *G6PC* | *GAA* |
| *GALC* | *GALE* | *GALK1* | *GALT* | *GARS* | *GBE1* | *GCH1* |
| *GCS1* | *GDAP1* | *GFPT1* | *GJB1* | *GJB3* | *GK* | *GLA* |
| *GLYCTK* | *GMPPB* | *GNB4* | *GNE* | *GPD1L* | *GYG1* | *GYS1* |
| *GYS2* | *HADH* | *HADHA* | *HADHB* | *HAMP* | *HARS* | *HCN4* |
| *HEXA* | *HFE* | *HFE2* | *HINT1* | *HK1* | *HNRNPA1* | *HNRNPA2B1* |
| *HNRNPDL* | *HNRPDL* | *HOXA1* | *HOXD10* | *HRAS* | *HSPB1* | *HSPB3* |
| *HSPB8* | *IFRD1* | *IGHMBP2* | *IKBKAP* | *INF2* | *ISCU* | *ISPD* |
| *ITGA7* | *ITGA9* | *KARS* | *KBTBD13* | *KCNA1* | *KCNE1* | *KCNE2* |
| *KCNE3* | *KCNH2* | *KCNJ16* | *KCNJ18* | *KCNJ2* | *KCNJ3* | *KCNJ5* |
| *KCNQ1* | *KHK* | *KIF1A* | *KIF21A* | *KIF5A* | *KLHL40* | *KLHL9* |
| *LAMA2* | *LAMB2* | *LAMP2* | *LARGE* | *LAS1L* | *LDB3* | *LDHA* |
| *LDHB* | *LDHC* | *LDHM* | *LIMS2* | *LITAF* | *LMNA* | *LPIN1* |
| *LRP4* | *LRSAM1* | *LYZ* | *MAPT* | *MARS* | *MATR3* | *MED25* |
| *MEGF10* | *MFN2* | *MGAT2* | *MLYCD* | *MMACHC* | *MOGS* | *MPDU1* |
| *MPI* | *MPZ* | *MSTN* | *MTAP* | *MTM1* | *MTMR2* | *MTO1* |
| *MUSK* | *MVK* | *MYBPC1* | *MYBPC3* | *MYF6* | *MYH14* | *MYH2* |
| *MYH3* | *MYH7* | *MYH8* | *MYLK2* | *MYOT* | *NAGLU* | *NDRG1* |
| *NEB* | *NEFL* | *NGF* | *NHLRC1* | *NIPA1* | *NTRK1* | *OPA1* |
| *ORAI1* | *OTC* | *PABPN1* | *PDK3* | *PDSS1* | *PDSS2* | *PEX1* |
| *PEX7* | *PFKM* | *PGAM2* | *PGK1* | *PGM1* | *PHKA1* | *PHKA2* |
| *PHKB* | *PHKG2* | *PHOX2A* | *PHYH* | *PIEZO2* | *PLA2G6* | *PLEC* |
| *PLEKHG5* | *PMM2* | *PMP22* | *PNPLA2* | *POLG* | *POMGNT1* | *POMGNT2* |
| *POMK* | *POMT1* | *POMT2* | *PREPL* | *PRKAG2* | *PRKG2* | *PRNP* |
| *PRPS1* | *PRX* | *PTPLA* | *PTRF* | *PYGL* | *PYGM* | *RAB7A* |
| *RAPSN* | *RBCK1* | *RBM28* | *REEP1* | *RFT1* | *RNF170* | *RYR1* |
| *SALL4* | *SBF1* | *SBF2* | *SCN11A* | *SCN1A* | *SCN1B* | *SCN3B* |
| *SCN4A* | *SCN4B* | *SCN5A* | *SCN9A* | *SCO2* | *SCP2* | *SECISBP2* |
| *SEPN1* | *SGCA* | *SGCB* | *SGCD* | *SGCE* | *SGCG* | *SGK196* |
| *SH3TC2* | *SIL1* | *SLC12A6* | *SLC22A5* | *SLC25A1* | *SLC25A12* | *SLC25A20* |
| *SLC25A3* | *SLC25A4* | *SLC2A2* | *SLC35A1* | *SLC35C1* | *SLC37A4* | *SLC40A1* |
| *SLC4A1* | *SLC5A7* | *SMCHD1* | *SMN1* | *SMN2* | *SNAP25* | *SOD1* |
| *SOX10* | *SPAST* | *SPG11* | *SPTLC1* | *SPTLC2* | *STIM1* | *SURF1* |
| *TALDO1* | *TAZ* | *TBX5* | *TCAP* | *TDP1* | *TFG* | *TGFB1* |
| *TH* | *TIA1* | *TK2* | *TMEM43* | *TMEM5* | *TNNI2* | *TNNI3* |
| *TNNT1* | *TNNT2* | *TNNT3* | *TNPO3* | *TNXB* | *TOR1AIP1* | *TPI1* |
| *TPM1* | *TPM2* | *TPM3* | *TRAPPC11* | *TRIM2* | *TRIM32* | *TRPV4* |
| *TTN* | *TTR* | *TUBB2B* | *TUBB3* | *UBA1* | *UTRN* | *VAPB* |
| *VCP* | *VMA21* | *VRK1* | *XK* | *YARS* | *ZBTB42* | *ZC4H2* |
