# Supplementary figures and images for: Clinical and genetic spectrum of sarcoglycanopathies in a large cohort of Chinese patients
Source: Orphanet J Rare Dis. 2019 Feb 14;14:43. doi: 10.1186/s13023-019-1021-9 (PMC6376703; doi:10.1186/s13023-019-1021-9)

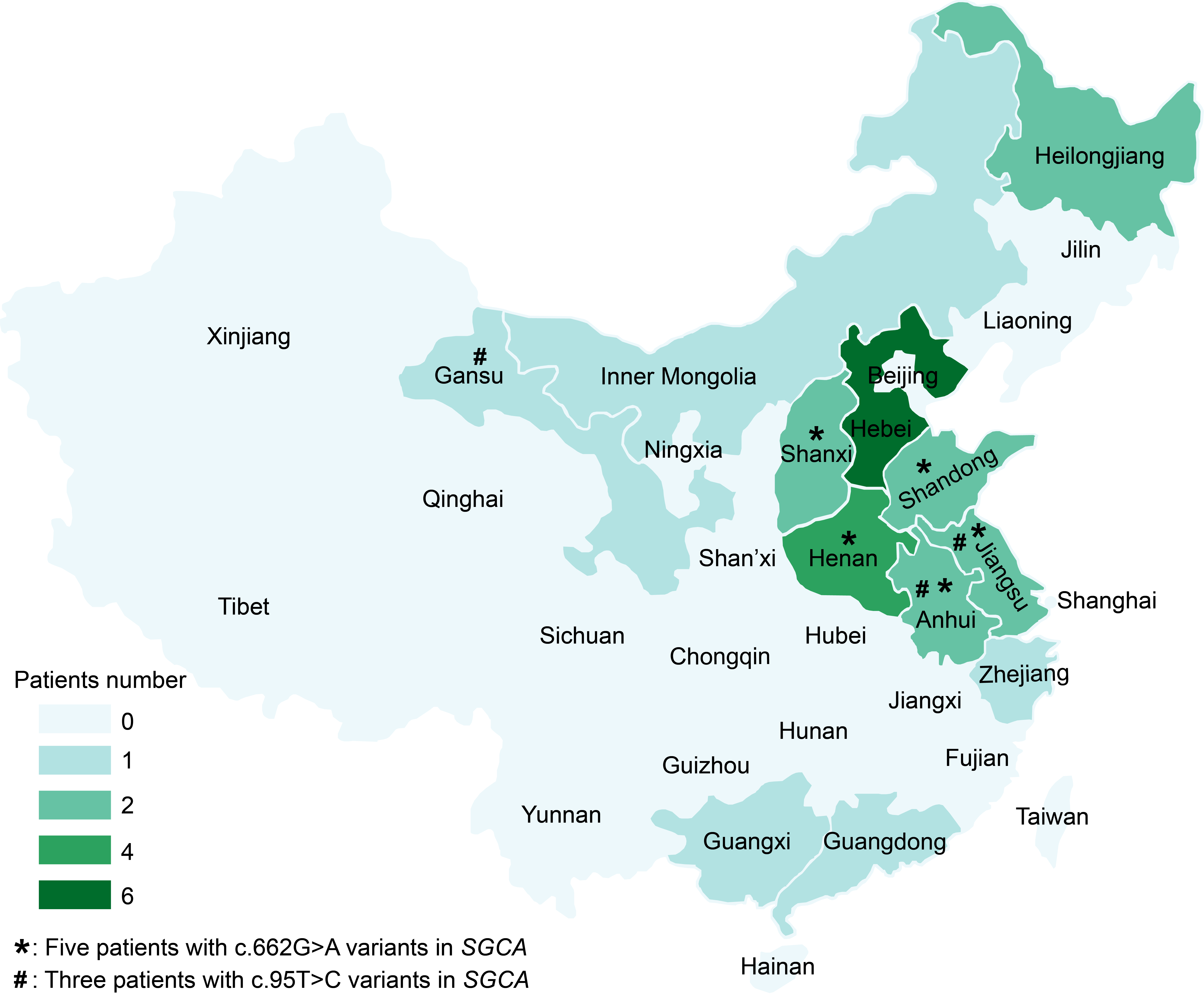

Supplement: Supplementary file 2 — Figure S1. Geographic origin of Chinese patients with sarcoglycanopathies and the common mutations identified in SGCA. (TIF 3913 kb) [file 13023_2019_1021_MOESM2_ESM.tif]

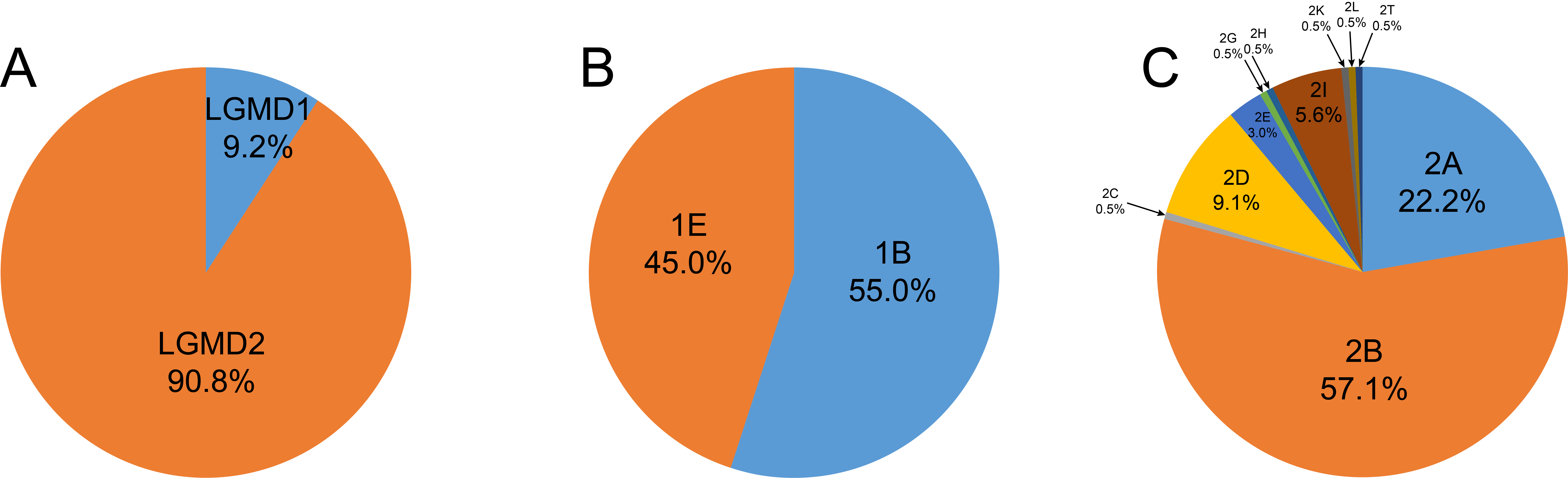

Supplement: Supplementary file 3 — Figure S2. The proportion of different LGMD subtypes. (A) Overview of LGMD subtypes, including autosomal dominant (LGMD1) or autosomal recessive (LGMD2) types. (B) LGMD1 comprised LGMD1E and LGMD1B. (C) The proportion of different LGMD2 subtypes. (TIF 2636 kb) [file 13023_2019_1021_MOESM3_ESM.tif]
