# Supplementary material for: Clinical and genetic spectrum of sarcoglycanopathies in a large cohort of Chinese patients
Source: Orphanet J Rare Dis. 2019 Feb 14;14:43. doi: 10.1186/s13023-019-1021-9 (PMC6376703; doi:10.1186/s13023-019-1021-9)
Supplement: Supplementary file 4 — Table S2. Nerve conduction study of patient 1. (DOCX 15 kb) [file 13023_2019_1021_MOESM4_ESM.docx]

Table S2. Nerve conduction study of patient 1.

|  | | Median nerve | Ulnar nerve | Peroneal nerve | Tibial nerve | Sural nerve | Superficial peroneal nerve |
| --- | --- | --- | --- | --- | --- | --- | --- |
| Motor nerves | Conduction velocity (m/s) | 19 | 21 | 23 | 22 | - | - |
|  | Distal amplitude (mV) | 6.667 | 1.616 | 0.658 | 5.016 | - | - |
| Sensory nerves | Conduction velocity (m/s) | 22 | 18 | - | - | 23 | 21 |
|  | Amplitude (uV) | 21.18 | 6.144 | - | - | 25.45 | 22.84 |

All the examined nerves were in the right limbs. mV, millivolts; uV, microVolt; m/s, meter per second.
