# Supplementary material for: Clinical and genetic spectrum of sarcoglycanopathies in a large cohort of Chinese patients
Source: Orphanet J Rare Dis. 2019 Feb 14;14:43. doi: 10.1186/s13023-019-1021-9 (PMC6376703; doi:10.1186/s13023-019-1021-9)
Supplement: Supplementary file 5 — Table S3. Pathologic changes and prediction of genotype based on expression of sarcoglycans in patients with sarcoglycanopathies. (DOCX 20 kb) [file 13023_2019_1021_MOESM5_ESM.docx]

Table S3. Pathologic changes and prediction of genotype based on expression of sarcoglycans in patients with sarcoglycanopathies.

| Patients | LGMD subtype | Gene | Histopathological pattern | α-SG | β-SG | γ-SG | Predicted  diagnosis |
| --- | --- | --- | --- | --- | --- | --- | --- |
| P1 | LGMD2D+CMT1A | *SGCA* | Dystrophic | 5 | 5 | 5 | Impossible |
| P2 | LGMD2D | *SGCA* | Mild myopathic changes | 3 | 1 | 1 | LGMD2D |
| P3 | LGMD2D | *SGCA* | Dystrophic | 4 | 2 | 1 | LGMD2D |
| P4 | LGMD2D | *SGCA* | Dystrophic | 5 | 5 | 3 | Impossible |
| P5 | LGMD2D | *SGCA* | Dystrophic | 4 | 4 | 4 | Impossible |
| P6 | LGMD2D | *SGCA* | Dystrophic | 5 | 5 | 3 | Impossible |
| P7 | LGMD2D | *SGCA* | Mild myopathic changes | 2 | 2 | 1 | Impossible |
| P8 | LGMD2D | *SGCA* | Mild myopathic changes | 2 | 1 | 1 | LGMD2D |
| P9 | LGMD2D | *SGCA* | Dystrophic | 5 | 4 | 3 | LGMD2D |
| P10 | LGMD2D | *SGCA* | Dystrophic | 4 | 4 | 3 | Impossible |
| P11 | LGMD2D | *SGCA* | Dystrophic | 5 | 5 | 3 | Impossible |
| P12 | LGMD2D | *SGCA* | Mild myopathic changes | 2 | 3 | 2 | LGMD2E |
| P13 | LGMD2D | *SGCA* | Dystrophic | 4 | 4 | 2 | Impossible |
| P14 | LGMD2D | *SGCA* | Mild myopathic changes | 2 | 2 | 1 | Impossible |
| P15 | LGMD2D | *SGCA* | Dystrophic | 5 | 5 | 3 | Impossible |
| P16 | LGMD2D | *SGCA* | Dystrophic | 4 | 5 | 3 | LGMD2E |
| P17 | LGMD2D | *SGCA* | Mild myopathic changes | 1 | 2 | 1 | LGMD2E |
| P18 | LGMD2D | *SGCA* | Dystrophic | 4 | 5 | 5 | Impossible |
| P19 | LGMD2E | *SGCB* | Dystrophic | 2 | 3 | 2 | LGMD2E |
| P20 | LGMD2E | *SGCB* | Dystrophic | 3 | 5 | 3 | LGMD2E |
| P21 | LGMD2E | *SGCB* | Dystrophic | 4 | 5 | 4 | LGMD2E |
| P22 | LGMD2E | *SGCB* | Dystrophic | 5 | 5 | 5 | Impossible |
| P23 | LGMD2E | *SGCB* | Dystrophic | 5 | 5 | 4 | Impossible |
| P24 | LGMD2E | *SGCB* | Dystrophic | 2 | 5 | 1 | LGMD2E |
| P25 | LGMD2C | *SGCG* | Dystrophic | 3 | 4 | 5 | LGMD2C |

Protein expression on sections were scored as follows: normal (score 1), slight reduction (score 2), reduction (score 3), severe reduction (score 4), and absence (score 5). LGMD, limb-girdle muscular dystrophy; CMT1A, Charcot-Marie-Tooth 1A; SG, sarcoglycan.
